# Enhancing Photodynamyc Therapy Efficacy by Combination Therapy: Dated, Current and Oncoming Strategies

**DOI:** 10.3390/cancers3022597

**Published:** 2011-06-09

**Authors:** Ilaria Postiglione, Angela Chiaviello, Giuseppe Palumbo

**Affiliations:** Department of Molecular and Cellular Biology and Pathology “L. Califano”, University Federico II, Via S. Pansini, Naples 5 80131, Italy; E-Mails: ilariapostiglione@intrefree.it (I.P.); angelachiaviello@libero.it (A.C.)

**Keywords:** photodynamic therapy, photosensitizers, combination therapy, targeted therapy

## Abstract

Combination therapy is a common practice in many medical disciplines. It is defined as the use of more than one drug to treat the same disease. Sometimes this expression describes the simultaneous use of therapeutic approaches that target different cellular/molecular pathways, increasing the chances of killing the diseased cell. This short review is concerned with therapeutic combinations in which PDT (Photodynamyc Therapy) is the core therapeutic partner. Besides the description of the principal methods used to assess the efficacy attained by combinations in respect to monotherapy, this review describes experimental results in which PDT was combined with conventional drugs in different experimental conditions. This inventory is far from exhaustive, as the number of photosensitizers used in combination with different drugs is very large. Reports cited in this work have been selected because considered representative. The combinations we have reviewed include the association of PDT with anti-oxidants, chemotherapeutics, drugs targeting topoisomerases I and II, antimetabolites and others. Some paragraphs are dedicated to PDT and immuno-modulation, others to associations of PDT with angiogenesis inhibitors, receptor inhibitors, radiotherapy and more. Finally, a look is dedicated to combinations involving the use of natural compounds and, as new entries, drugs that act as proteasome inhibitors.

## 1. Introduction

The fundamental rationale for combination therapy in cancer is to use approaches that work by different mechanisms of action. Combined treatments (two or more approaches) may target different key signal transduction pathways and may be more efficient in destroying cancer cells and in eluding the cellular resistance mechanisms. Besides this, another evident advantage of combining different approaches may be an enhancement in overall therapeutic efficacy. The combination may in fact give additive or even synergistic effects, so that a reduction of the dose of the most toxic component is sometimes feasible, with elimination or at least lessening of noxious side effects.

This work, without any presumption of completeness, is an attempt to review some of the experimental results that have been obtained when PDT has been combined with traditional or innovative cures.

Photodynamic therapy is an approved therapeutic approach for the management of a variety of specific types of tumors and several precancerous and non cancerous diseases. This therapy requires a photosensitizer (*i.e.*, a light-absorbing compound) and a light source that emits at suitable wavelength that matches the absorption peak of the photosensitizer [1]. Some of the most popular photosensitizers that have been approved for use in humans are indicated in Table 1 (incomplete list).

The effectiveness of PDT depends on the production of reactive oxygen species within the cell that are generated through two types of photoreactions, indicated as Type I and Type II reactions. Type I involves electron/hydrogen transfer directly from the photosensitiser, producing ions, or electron/hydrogen extraction from a molecule to form free radicals. These radicals preferentially react with oxygen, resulting in the rapid production of highly reactive oxygen species. These radicals then attack cellular targets [2]. Type II reaction transfers the photosensitizer's energy excess directly to oxygen (triplet) producing singlet oxygen [3]. This species plays a prominent role in PDT-mediated cell damage [4]. Several observations have suggested that the efficiency of a photodynamic treatment strongly depends on the type, concentration and intracellular localization of the photosensitizer. Also light wavelength, light fluence and fluence rate are important to ensure sufficient oxygen availability and supply.

Reactive oxygen species (ROS) have been shown to destroy tumors by several different mechanisms [5,6]. Indeed PDT may:

- Directly kill tumor cells. This may occur through necrosis or apoptosis mechanisms [7];
- Induce alterations in the tumor vasculature leading to microvascular shutdown and hypoxia [8];
- Induce inflammatory and immune responses [9,10,11].

When the interplay of these components occurs efficiently, long term tumor control is possible.

**Figure 1.**
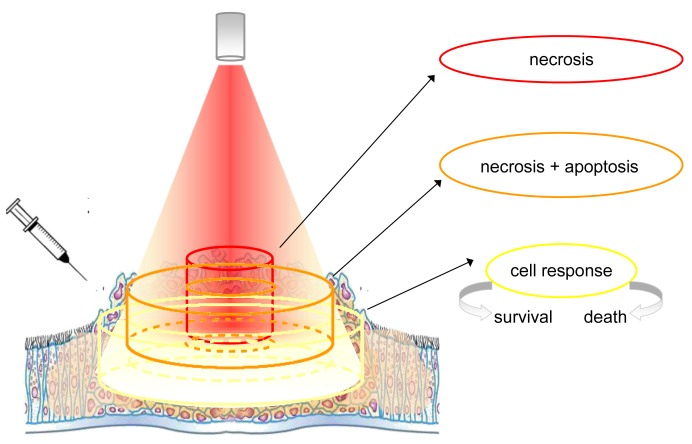
Over-simplified picture of light distribution and cellular responses during PDT.

The topically or systemically administered photosensitizers accumulate preferentially within cancerous tissues, but the selective concentration within the cancerous cells is only ideal. In fact, several factors such as the extent of vascularization, the type of photosensitizer and others, affect this unbalanced distribution. Photosensitizers are activated by exposure to light (Figure 1).

Albeit the figure is representative of the light distribution produced by a source equipped with an optical fiber terminating with a microlens, however, the concept can be extended also to other situations in which the distribution of light is different (*i.e.*, that produced by a cylindrical diffuser). In any case, the energy delivered is never equally distributed, being maximal at the centre and minimal at the borders of the illuminated area. In addition, the amount of light that penetrates the tissue decreases rapidly and the lower cell layers receive less and less energy. Cells are exposed to quantities of energy that depend on the relative position and distance from the irradiating light beam. For simplicity we can distinguish 3 cylindrical zones (from the centre to the edges): The first contains cells that are directly exposed to the light beam, and absorb the highest quantity of energy. The greatest effects are achieved in the tissue that receives the highest light fluence, but only if there is sufficient photosensitizer and oxygen (this depends on how well vascularized that part of the tissue is and whether the fluence rate is such that the tissue does not become hypoxic). In these conditions, most of these cells, being intensely damaged, proceed rapidly to necrosis. A second, more external zone contains cells that receive a lesser dose of light, either because they are at the periphery of the light beam or because they are localized in a layer not immediately near to the surface. In this event, the damage may be yet significant, but certainly less intense than in the previous case. Although most cells still proceed to necrosis, a significant fraction of them may also activate an apoptotic process. The third zone comprises all cells that are in very peripheral positions but, because they absorb some light, are capable of some photosensitization. In this event, the mild, non-extensive photoactivation is not able to kill the cells directly but rather elicits unpredictable effects. The study of these non-lethal conditions has provided valuable information on the cell reaction to PDT. Under these conditions, in fact, the spared cells elicit cellular and molecular responses, whose characterization is the premise necessary to improve photodynamic-based treatments, including those in combination with other therapies.

Until now, many strategies have been proposed to maximize and potentiate the therapeutic effects of photodynamic therapy when used in combination with other curative partners.

**Figure 2.**
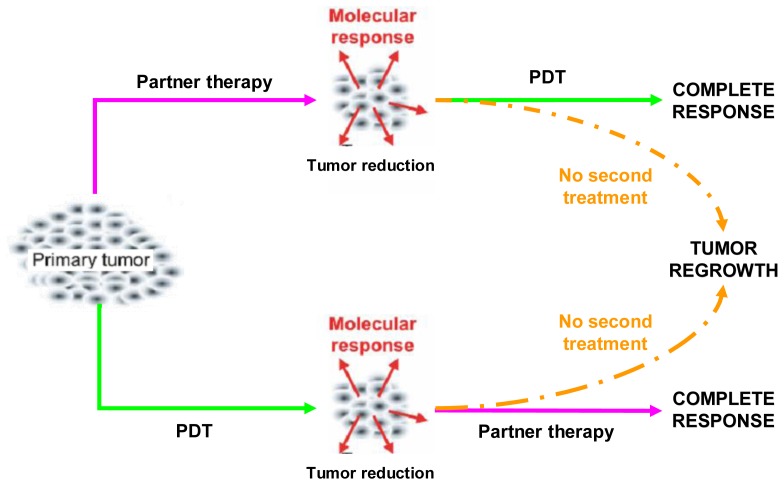
Combined therapy approaches.

Combination regimens, that include PDT and a partner treatment, should be aimed at increasing the therapeutic effectiveness and, above all, at eradicating the tumor completely. In principle, this may be achieved either by counteracting the prosurvival signaling triggered in tumor cells that resisted PDT or, alternatively, by pre-weakening the tumor cells so that they become more susceptible to a later PDT treatment (Figure 2) [12].

Our experience in the field is about ten years old. We first showed that PDT was affecting the cell cycle [13] and, later, that combination therapy was particularly effective when the partner approach was aimed at targeting “different” critical cellular pathways [14]. Synergistic effects could only be obtained under these conditions. This hypothesis was confirmed by numerous successive observations [15].

## 2. Assessing Efficacy of Combined Therapy

The efficacy of a combined therapy is established on a simple empirical basis. However, there are at least two systematic methods of evaluating how effective a combination is. They are: The graphic isobologram method and a mathematical method based on a simple equation that calculates a representative index, known as Combination Index.

### 2.1. Isobolographic Method

Isobolograms are constructed according to a “fixed dose method” [16]. First, the responses in a fraction *x* of the cells are evaluated after each individual treatment. A fraction corresponding to x% response is established as suitable reference point and indicated as ED*_x_*. According to the combination approach, two different curves are assembled, maintaining a constant concentration of the first component and varying the second one, or *vice versa*. This allows us to experimentally obtain the dose of one component that is necessary to attain the prefixed effect in the presence of an established concentration of the second one (and *vice versa*).

In a typical isobologram, the ED*_x_*, computed by individual dose-response curves, is plotted on the vertical and horizontal axes, respectively. The theoretical dose-additive line, including its 95% fiducial limits, is attained by joining the two chosen ED*_x_*s. If the experimental ED*_x_* of the combination is within the boundary of the dose-additive line and its confidence interval (*i.e.*, the experimental point is nearly coincident with the theoretical point), then the specific combination exerts a dose-additive effect. The effect is synergistic if the experimental point and theoretical point are below the boundaries and antagonistic if they are above the boundaries.

### 2.2. Combination Index

The interaction (namely synergism, additivity and antagonism) of different drugs in combination has often been quantitatively estimated through the algorithm proposed by Chou and Talalay [17].

This algorithm calculates a numerical index, namely Combination Index, the value of which provides direct information on additivity, antagonism or synergism that derive from specific therapeutic combinations.

The Combination Index may be expressed according to the following simplified relation:

$$\text{CI}={\left(\text{D}\right)}_{1}/{\left(\text{Dx}\right)}_{1}+{\left(\text{D}\right)}_{2}/{\left(\text{Dx}\right)}_{2}+{\left(\text{D}\right)}_{1}\;{\left(\text{D}\right)}_{2}/{\left(\text{Dx}\right)}_{1}{\left(\text{Dx}\right)}_{2}$$

where (D)_1_ and (D)_2_ are the doses of drug 1 and 2 which are required to produce x% effect in combination, while (Dx)_1_ and (Dx)_2_ are the dose of drug 1 and drug 2 required to produce the same x% effect individually.

Thus, for agents that have independent modes of action, CI < 1, CI = 1, and CI > 1 indicate synergy, additive effect and antagonism, respectively.

Specific applications of combination index and isobolographic analyses to PDT used in combination with other more traditional therapeutic approaches (*i.e.*, cisplatin) are reported by Varriale *et al.* [13] and Crescenzi *et al.* [14].

## 3. PDT in Combination Therapy

The next paragraphs report some of the rather numerous applications of combined therapy in which PDT has been associated with both traditional and innovative therapeutic approaches for cancer treatment. The description contains various examples, but does not claim completeness.

### 3.1. Anti-Oxidant Agents

As repeatedly mentioned, PDT kills cells through intense and localized generation of reactive oxygen species. The presence of radical scavengers and/or antioxidants should nullify or counteract the effects of PDT. Therefore, a combination of antioxidants, which are considered chemopreventive agents against cancer [18,19] with PDT, appears rather unconvincing. Nonetheless, a number of reports contradict this affirmation, possibly because, as widely reported, anti-oxidants may sometimes reveal unexpected pro-oxidant properties. With regard s̶ to this issue, Buettner and co-workers [20], for example, demonstrated that, in the presence of metal traces (in their case iron), ascorbate combined with Photofrin/PDT enhanced the production of radicals and decreased cell survival of various cell lines. A cooperative therapeutic outcome was also observed in other systems and other conditions when ascorbate was associated with other photosensitizers. Various interpretations and explanations have been reported in this regard. According to some, the effects associated with the combination ascorbate + *5* − *ALA*/PDT in rat DS-sarcoma cancer cells, were once again attributable to pro-oxidant properties of ascorbate only when its concentration was kept very low [21]. Other authors, studying the effects of the combination with benzoporphyrin derivative/PDT in HL60 cells, explained the synergistic therapeutic outcome on the basis of a cascade of effects following ascorbate reaction with singlet oxygen to form hydrogen peroxide. This species, stimulating myeloperoxidase activity, generates more toxic oxidant species. Thus, they concluded that the addition of ascorbate to cells expressing high myeloperoxidase levels followed by photosensitization would strongly enhance the toxicity of the photodynamic action due to the augmented formation of highly diffusible hydrogen peroxide and other toxic radicals [22].

Several other limited observations have been reported regarding the successful use of other antioxidants in association with PDT. For example, it has been also observed that the combination of the antioxidant agent butyl-4-hydroxyanisole and HpD/PDT on Ehrlich ascites carcinoma cells may combine in a wide range of positive therapeutic effects spanning from additive to synergistic [23]. Melnikova *et al.* [24] studying HT29 adenocarcinoma cells and MRC-25 normal fibroblasts, demonstrated that the efficacy of m-tetrahydroxyphenylchlorin mTHPC/PDT could be synergistically enhanced in the presence alpha-tocopherol, but only when the vitamin was present at elevated concentrations. The same authors, using a water-soluble alpha-tocopherol analogue in combination with mTHPC/PDT demonstrated a remarkable reduction in tumor growth in an *in vivo* model (HT29 xenografts in nude mice), however, only when the analogue, namely Trolox, was administered to mice prior to PDT [25].

In conclusion, it appears that the final therapeutic outcome determined by the use of antioxidants in association with PDT is dependent on many variables or conditions and on the selected model systems. Besides the nature, concentration and localization of the photosensitizer, the following factors also seem particularly important: The anti-oxidant concentration, the presence of catalytic trace metals, the order and the time interval between the administration of the drug and the light exposure, the light fluence, the oxygen accessibility and more.

### 3.2. Chemotherapeutic Agents

Chemotherapeutic agents can be divided into two large categories according to their direct or indirect effect on DNA. The group of agents that directly targets DNA is composed of alkylating agents, antitumor antibiotics and inhibitors of topoisomerases. The following sections are concerned with some of these drugs that have found application in combination with PDT.

#### 3.2.1. Alkylating agents

Cisplatin and its derivatives (oxaliplatin and carboplatin) are commonly used drugs to treat different neoplasm, including sarcomas, lymphomas, small cell lung and ovarian cancers [26]. However, their good clinical efficacy is often limited by severe adverse toxic effects, as these drugs, lacking cancer selectivity, do not spare the normal tissues [27,28].

Several papers have described the study of these drugs in combination with PDT. For example, clearly positive results have been reported in experiments exploiting the combination of Photofrin/PDT with cisplatin for efficient killing of mouse lymphoma cells [29] or esophageal carcinoma cells where an enhanced cytotoxic and apoptotic effect was demonstrated [30].

Some effort in this direction has also been made in our laboratory. In particular, we investigated the effects of the combination of low-dose cisplatin with indocyanine green/PDT on breast cancer cells. Viability and metabolic data demonstrated mutual reinforcement of therapeutic efficacy. In particular, we showed that the favorable effects of this combined treatment are due to actions exerted separately by each approach on cells in different phases of the cycle [14].

A newer approach for the combination of PDT and cisplatin has been also offered by Lottner *et al.* [31]. These authors have synthesized different hematoporphyrin-based platinum derivatives bearing a phototoxic ligand, so that it was possible to join the intrinsic cytostatic activity of cisplatin (or oxaliplatin) to the photodynamic effect of hematoporphyrin in a single molecule. The authors evaluated the cytotoxicity and phototoxicity of some of these derivatives against bladder cancer and normal urothelial cells, demonstrating a remarkable antiproliferative and selective effect compared to cisplatin and hematoporphyrin alone or a combination of the drugs.

Carboplatin, a less nephrotoxic analogue of cisplatin, has been employed in combination with 9-hydroxypheophorbide alpha (9-HPbD)/PDT to treat head and neck cancer cell lines *in vitro*. In these experimental systems enhanced cytotoxic and pro-apoptotic effects have been reported [32].

All the findings regarding the association of cisplatin (or its derivatives) with a photodynamic treatment conclude unanimously that the combined modality often results in synergy. This fact is obviously important as it implies the possibility of lowering the dose of the inevitably toxic antineoplastic drug without sacrificing overall therapeutic efficacy.

#### 3.2.2. Antitumor antibiotics

##### 3.2.2.1. Doxorubicin

Among the antitumor antibiotics, doxorubicin is commonly used in the treatment of a wide range of cancers such as hematological malignancies, carcinomas, and soft tissue sarcomas. This drug, besides being a DNA intercalating molecule and topoisomerase II inhibitor, probably exerts many other antitumor activities through alternative and complex modes of action [33,34].

Casas *et al.* [35] evaluated the interaction between *5*-*ALA*/PDT and doxorubicin in mice bearing transplantable mammary adenocarcinomas. Tumor explants of doxorubicin-treated mice were first subjected to 5-ALA/PDT *in vitro* and then re-implanted into test animals that showed that inhibition of tumour growth was significantly enhanced by the combined treatment. The authors assigned the observed enhancement of PDT to the weakening of cellular defense mechanisms by the pre-treatment involving free radical generation by doxorubicin.

Canti *et al.* [36] investigated the effects of the combination of disulfonated aluminum phthalocyanine (AlS2Pc/PDT) and doxorubicin on mice bearing murine leukemia and lymphoma. Low chemotherapy doses were ineffective, but the combination of doxorubicin and AlS2Pc/PDT had a significantly additive antitumor effect.

Shiah *et al.* [37] demonstrated the selective tumor targeting and the antitumor efficacy of the association of chemotherapy (*N*-(2-hydroxypropyl)methacrylamide (HPMA) copolymer-bound doxorubicin) and mesochlorin e6 monoethylenediamine (Mce6)/PDT in nude mice bearing human ovarian OVCAR-3 carcinoma xenografts.

The cytotoxic and antitumor effects of doxorubicin in combination with mTHPC)/PDT have also been verified both *in vitro* (murine hepatoma cells) and *in vivo* (murine liver) [38].

Finally, the anticancer efficacy of doxorubicin in combination with methylene blue/PDT has been investigated in a drug-resistant mouse tumor model [39]. In this case, additional novelty was provided by the use of surfactant-polymer hybrid nanoparticles for synchronized delivery of the two drugs. Nanoparticle-mediated combination treatment resulted in enhanced tumor accumulation of both doxorubicin and methylene blue, significant inhibition of tumor cell proliferation, increased induction of apoptosis and improved animal survival.

##### 3.2.2.2. Mitomycin C

Mitomycin C is an anti-tumor antibiotic that inhibits DNA synthesis [40]. The group of Ma investigated the cytotoxic effects of mitomycin C in human colon adenocarcinoma cell lines and then compared this treatment with a combination treatment involving Photofrin/PDT [41,42]. The authors observed that the combined treatment was particularly effective, yielding curative responses from additive to synergistic, especially at higher antineoplastic drug concentration. Similar results were obtained in mouse fibrosarcoma [43] and rat colon carcinoma implanted in syngenic animal models [44]. Although each treatment alone induced a small tumor growth delay, the combination was significantly more effective.

In addition to Photofrin, mitomycin C has been also successfully used in combination with m-THPC) and bacteriochlorin a in animal models of fibrosarcoma [45]. In addition the *5*-*ALA*/PDT has been employed in combination with mitomycin C. This combination was very effective when used to treat bladder cancer cell lines, including cells that were notoriously resistant to mitomycin. On the basis of these findings, the authors suggested that the combination of mitomycin C and *5*-*ALA*/PDT in the treatment of superficial bladder tumors that have recurred despite intravesical cytotoxic drug treatment should be considered a workable therapeutic approach [46]. A phase-1 study performed on patients affected with recurrent superficial bladder cancer demonstrated the safety of mitomycin C in combination with 5-ALA/PDT, and its potential for the management of difficult-to-control superficial cell carcinoma and carcinoma *in situ* of the bladder [47].

#### 3.2.3. Drugs targeting Topoisomerases

Topoisomerases (I and II) are enzymes needed to modify DNA's topological structure by unwinding and winding filaments during transcription and replication [48]. In recent years, these enzymes have been recognized as excellent targets for cancer chemotherapy as their inhibition interfering with the cell cycle causes DNA single and double strand breaks, impairs genome integrity and finally induces apoptosis and cell death [48]. To date, topoisomerase inhibitors are considered among the most active anticancer agents.

##### 3.2.3.1. Topo I inhibitors

The quinoline alkaloid camptothecin (CPT) is a potent inhibitor of topoisomerase I (topo I). It shows a remarkable anticancer activity that unfortunately goes along with high toxicity. Two analogues of this molecule, namely topotecan and irinotecan [49,50], have been recently satisfactorily introduced for therapy in humans being endowed of reasonable toxicity. Although neither one of these compounds has been used as such in combination with PDT, Peng *et al.* [51] quite recently introduced chlorin-core star-shaped block copolymer (CSBC) micelles loaded with an active metabolite of irinotecan (7-ethyl-10-hydroxy-camptothecin). These micelles, carrying both the photosensitizer and the antineoplastic agent, were profitably used *in vitro* and *in vivo*. *In vivo*, it has been shown that the 7-ethyl-10-hydroxy-camptothecin/CSBC micelles preferentially accumulated in tumor tissues, assuring higher specificity and response. *In vitro*, the combination indicated that the approach could result in synergistic effects. In addition to positive effects of the combination *per se*, these data suggest that micelle-based delivery systems may further improve the combination strategy.

##### 3.2.3.2. Topo II inhibitors

Based on their mechanism of action, topo II inhibitors have been classified on the basis of their intercalating and non intercalating properties [52].

Etoposide is a non intercalating agent that forms a cleavable complex with DNA and is one of the most active antitumor drugs against solid tumors [48]. The use of this drug with PDT has been proposed by Gantchev *et al.* [53]. The authors compared the effects of individual toxicities of etoposide and PDT with aluminum phthalocyanine tetrasulfonate (AlPcS4), with that of their combination in human leukaemia cells. This combination was very effective, as indicated by the observed strong growth inhibition, significant loss of clonogenicity potential, cell cycle arrest and DNA fragmentation.

#### 3.2.4. Antimetabolites

The group of chemotherapeutic agents that indirectly target DNA is comparable to antimetabolites that interfere with synthesis and replication of DNA. Such an effect is accomplished by DNA base analogues or through inhibitors of specific enzymatic activities necessary to accomplish correct DNA synthesis.

Regarding DNA base analogues, there are some reports that combine gemcitabine with PDT. Gemcitabine is a deoxycytidine analogue effective against solid tumors, including non-small cell lung cancer [54]. Data previously obtained in our laboratory indicated that the combination of Photofrin/PDT and gemcitabine caused an additive effect in adenocarcinoma lung cancer cell lines [55]. Similar results were reported more recently in a study that demonstrated the particular efficacy of Photosan/PDT and gemcitabine in curing nude mice bearing human pancreatic cancer [56].

Methotrexate, a known inhibitor of DNA synthesis has been frequently employed in combination with PDT. Methotrexate is a structural analogue of folic acid and a potent inhibitor of dihydrofolate reductase. It potently interferes with the synthesis of thymidylate and purine nucleotides and hence inhibits tumor progression [57].

A very effective therapeutic combination associates this drug with 5-ALA/PDT. This combination has been shown to cause a synergistic cytotoxic effect in human prostate carcinoma cells [58] and epithelial squamous carcinoma models both *in vitro* and *in vivo* [59]. Interestingly, the differential and selective response is based on the methotrexate-mediated induction of mitochondrial coproporphyrinogen oxidase (CPO) expression that is particularly elevated in malignant cells. Hence, whatever the amount of 5-ALA administered and taken up by the cells, pre-treatment with methotrexate (that stimulates CPO, the major enzyme for protoporphyrin synthesis), promotes a hyperproduction of the endogenous photosensitizer PpIX. Extra production of PpIX is also apparent when methotrexate is used at lower doses. This fact is important as it allows the dose of the toxic methotrexate to be lowered and, yet, renders PDT more effective, because of the increase in PpIX production.

#### 3.2.5. Drugs targeting the cytoskeleton

Many chemotherapeutic drugs act on the cytoskeleton, preventing the progression of the cell cycle. The most popular mitotic inhibitors in cancer therapy include Vinca alkaloids and Taxanes.

*Vinca alkaloids*—The vinca alkaloids are amines of natural origin. They inhibit microtubule depolymerization, thereby affecting cell mitosis. In particular Vincristine and Vinblastine are used to treat leukaemia, lymphoma, lung and breast cancer, while Vinorelbine, a semi-synthetic alkaloid, is indicated specifically in the treatment of breast cancer and non-small-cell lung cancer.

Quite recently, Ma *et al.* [60] demonstrated that the combination of meso-tetra-(di-adjacent-sulphonatophenyl)-porphine/PDT and Vincristine enhanced overall antitumor activity against mammary murine cancers, provided that PDT was administered within a defined (and narrow) time window.

Vinblastine has been tested in combination with Photofrin/PDT both *in vivo* and *in vitro* models of ovarian cancers [61]. In both systems, the combination protocols yielded positive results in that the anti-neoplastic effect was enhanced while cytotoxicity was reduced because of the lower Vinblastine dose needed.

Taxanes are complex terpenes produced by plants of the genus Taxus. When used as drugs (Paclitaxel and Docetaxel), their principal mechanism of action consists of the disruption of microtubule function by stabilizing microtubule formation, thereby stopping cellular division.

Paclitaxel and its semi-synthetic derivative Docetaxel are two drugs frequently used in cancer therapy (particularly lung, ovary, breast, Kaposi's sarcoma and other) [62,63]. These drugs have been employed in several experimental systems in combination with PDT, with gratifying results. For example, Park *et al.* found that Paclitaxel enhanced the cytotoxic effect of Verteporfirin/PDT on gastrointestinal human tumor. In particular, these authors observed that cytotoxicity induced by PDT was markedly potentiated by pre-treatment of cells with Paclitaxel at low doses. They reported also that cell death occurred through an apoptotic mechanism with a significant mitochondria cytochrome c release, independent of Bax or Bid activation [64].

According to another observation [65], the association of PDT with Paclitaxel has additional positive features in that the combination seems to overcome tumor cell resistance against the drug. The problem of Paclitaxel resistance can apparently be solved by another type of association. Indeed, it has been demonstrated [65] that the combination of the protein kinase C (PKC) inhibitor calphostin C with PDT potently kills breast tumor cells resistant to Paclitaxel. The mechanism by which this resistance is overcome requires the induction of cytoplasmic vacuolization without activation of typical apoptotic pathways. Consequently, it has been suggested that calphostin C may prove useful clinically to combat tumor growth in breast cancer patients particularly in association with PDT.

### 3.3. Immunotherapy

The ideal cancer therapy would destroy the primary tumor and trigger the immune system to recognize and eradicate any residual tumor cells, both at the site of the primary tumor and at metastases. If this does not occur, cancer cells escape immune control allowing neoplasias recurrence [66,67].

Immunological approaches used to potentiate PDT, in general, can be divided into non-specific and specific methods, depending on whether the immune system directly or indirectly affects cancer progression.

Non-specific methods are based on the administration of substances that influence, regulate and boost the overall activity of the immune system. Specific methods, in contrast, exploit the presence of malignant cell-associated antigens which should be specifically recognized by cellular and humoral effectors of the immune system. Photoimmunotherapy (PIT) and PDT supported by a specific immunotherapy have been classified as specific methods [68].

Obviously, the combination of PDT and immunotherapy should be aimed at sustaining and amplifying immune system response against the cancerous cells. To this purpose, several strategies have been developed. They include approaches aimed at upregulating leukocyte adhesion molecules, potentiating neutrophil and macrophage recruitment or inducing secondary cytokines, activating dendritic cells, CD4^+^ helper T-lymphocytes, B lymphocytes and natural killer cells, sensitizing CD8^+^ cytotoxic T-lymphocytes, downregulating CD4^+^CD25^+^ T-regulatory cells and inactivating tumor cells through the so-called adaptative immunity [69].

#### 3.3.1. Immunomodulation

It is generally acknowledged that PDT leads to local inflammation and invasion of the tumor by immune cells [70-72]. This aspect suggests the possibility of potentiating the immune response by supporting this process with the help of suitable immuno-stimulators. In this way, the recruitment of neutrophils and macrophages is highly amplified and the assault against the cancerous cells may be significantly enhanced.

Granulocyte-macrophage colony stimulating factor (GM-CSF) and granulocyte colony stimulating factor (G-CSF) are endogenous cytokines that regulate granulocyte functions and play major roles in the stimulation of granulopoiesis in the bone marrow [73]. Experimental proof that G-CSF improves the efficacy of PDT was obtained by various authors. Krosl *et al.* [74] reported curative effects for Photofrin and benzoporphyrin derivative (BPD)/PDT in mice bearing a genetically modified murine squamous cell carcinoma cells (SCCVII) producing GM-CSF. Similarly, Golab *et al.* showed that the intratumoral injection of recombinant human G-CSF in association with Photofrin/PDT was remarkably effective against colon adenocarcinoma and Lewis lung carcinoma in tumor-bearing mice. Treatment with GM-CSF resulted in higher cytotoxic activity of tumor-associated macrophages against SCCVII cells [75].

The combination of PDT with an immuno-adjuvant agent has also been applied in humans to patients affected with Bowenoid papulosis [76]. In pilot study, the photodynamic treatment was based on *5*-*ALA*, while the immuno-modifier agent used was Imiquimod (3-(2-methylpropyl)-3,5,8-triazatri-cyclo[7.4.0.0^2,6^]trideca-1(9),2(6),4,7,10,12-hexaen-7-amine) a drug that stimulates the immune response through the induction of cytokines. Until now, this type of approach has found frequent use in treatment of actinic keratosis, superficial basal cell carcinoma, and external genital and perianal warts [77]. Although a low rate of recurrence and even complete response have been claimed in large a fraction of the patients, the real effectiveness of Imiquimod immunotherapy in combination with PDT has yet to be definitively established.

Macrophage-activating factors have been applied with promising results in therapy [78]. In this regard, Photofrin/PDT has been combined with the specific macrophage-activating factor (DBPMAF) and resulted in cure of a squamous cell carcinoma in a murine model [79].

One of the mechanisms by which the innate immune system senses the invasion of pathogenic micro-organisms takes advantage of the Toll-like receptors (TLRs), which are expressed on the surface of monocytes, macrophages, dendritic cells, mast cells and some epithelial cells, recognize specific molecular patterns present on the surface of many microbes [80-82]. These receptors are sensors providing early warnings of infection. Their activation can induce the expression of NF-κB and, consequently, of other genes involved in the start of the anti-tumor immune response [83]. Consequently, it has been logically hypothesized that the administration of immunoadjuvants (as the components of bacterial cells that are among the most active TLR ligands) together with suitable PDT regimens might be an effective combinatorial approach to fight cancer [84,85]. In this regard an estimation of non-specific immunotherapeutic approach associated with PDT has been attempted by Korbelik *et al.* [86]. These authors tested PDT with BPD in association with various adjuvant agents such as Zymosan, γ-inulin and INF-γ. Zymosan (a cell wall preparation of *Saccharomyces cerevisiae*), and γ-inulin (a carbohydrate derived from the Compositae plant family) are potent activators of the complement, while INF-γ is a cytokine endowed of with anti-tumor, antiviral and immunoregulatory properties [87]. The combination protocol was used to treat a highly malignant mouse melanoma that is one of the most PDT-resistant tumors. The results of such investigations were very promising as the times for tumor recurrence in treated mice were at least three times longer than those treated with PDT alone. In addition, the same authors tested the association of PDT with γ-inulin *in vitro* on two transplantable fibrosarcoma cell lines. The effectiveness of this treatment was remarkable as the recovery of the tumor transplanted mice, according to their findings, was complete (100%). Indeed, γ-inulin is possibly the most promising complement activator, compared with other activators like Zymosan, streptokinase and urokinase, as inulin-based adjuvants are very effective and do not present serious side effects [88]. At variance, Zymosan, although equally effective *in vivo*, may be accompanied by important side effects like acute peritonitis and multiple organ failure [89].

Several immunoadjuvants have bacterial or fungal origin. Some of these have been employed in association with PDT. An immunoadjuvant agent prepared from *Corynebacterium parvum* has, for example, been tested in combination with haematoporphyrin derivative (HPD)/PDT to treat subcutaneous bladder cancer in mice [90]. Significant therapeutic efficacy was observed when PDT was followed by the administration of high doses of the agent. Similarly, Bacille Calmette-Guerin (BCG), an immunoadjuvant employed for many years for the therapy of superficial transitional cell carcinoma of the urinary bladder, has been used in combination with PDT. This treatment resulted in a significant delay in re-growth of an experimental mammary sarcoma in animals [91,92]. Unfortunately, as the use of BCG is not devoid of important side effects, this strategy appears to be of limited importance [93]. Another bacterium-derived immunostimulant that merits mention is OK432, a heat and penicillin G treated lyophilized powder of the Su-strain of *Streptococcus pyogenes*. OK-432 has been tested in combination with HpD/PDT in mice bearing NR-S1 mouse squamous cell carcinoma. Even if no animals fully recovered from the tumor, their survival time was significantly prolonged when OK-432 and PDT were combined. The beneficial effects were especially observed when OK-432 was injected intratumorally before PDT [94]. Finally, a study has been reported in which Photofrin/PDT was combined with a potent immunity inducer of fungal origin extracted from the polysaccharide Schizophyllan. The combination was successfully employed to cure aggressive squamous-cell carcinoma transplanted in nude mice [95]. Similar results have been reported by Chen and colleagues [96,97] that showed that a preparation of glycated chitosan derived from shrimp shells injected intratumorally significantly increased the curative effects of Photofrin/PDT on experimental mammary sarcomas and lung tumors.

Another approach focuses attention on potentiating the cellular component of the anti-tumor immune response targeting the immunosuppressive CD4^+^CD25^+^ T-regulatory cells. This has been exploited by Castano *et al.* [98] combining PDT with low dose cyclophosphamide (CY). In fact, it has been demonstrated that CD4^+^CD25^+^ T-regulatory cells are depleted by a low dose of cyclophosphamide, thus potentiating the immune response. The combination of cyclophosphamide with BPD/PDT for treatment of a metastatic murine tumor model led to a significant number of long-term cures and resistance to tumor re-challenge, whereas each treatment alone led to 100% death from progressive tumors or metastasis [99]. The examination of splenocytes recovered from tumor-bearing mice after low dose CY showed that CD4^+^CD25^+^ T cells were reduced in number, and the splenocytes secreted significantly less transforming growth factor-β (TGFβ),an important immunosuppressive cytokine secreted by T-regulatory cells, while stimulating the same cells [100,101].

#### 3.3.2. Photoimmunotherapy

Photoimmunotherapy is based on photosensitizers conjugated with monoclonal antibodies (mAbs) (or their fragments) that specifically target antigenic determinants exposed on tumor cells. Several photosensitizers (e.g., AlPcS4, mTHPC, pheophorbide a, chlorin e6, BPD) linked to tumor-specific monoclonal antibodies (such as C225, U36, 425, E48, HER50, HER66 mAbs) have found some application in photoimmunotherapy [102-106]. Although this approach has been given major credit among specialists, final protocols have not yet been unanimously established. In addition, several drawbacks exist as there are important technical problems associated with chemical coupling, the reduced phototoxicity of the complexes and their limited penetration into poorly vascularized tumors.

#### 3.3.3. Adoptive immunity

The term adoptive transfer applies to all therapies that consist of the transfer of components of the immune system that were, in advance, made proficient in arising a specific immune response. Also such an approach has been used in combination with PDT [107,108].

Jalili *et al.* [109], performing Photofrin/PDT on murine colon adenocarcinoma cells *in vitro*, described the induction of apoptotic and necrotic cell death and overexpression of specific protein antigens. Immature dendritic cells co-cultured with these cells acquired functional features of maturation and activation. The inoculation of such cells into mice bearing PDT-treated colon adenocarcinoma tumors resulted in effective anti-tumor responses, including decreased tumor size.

Another example of combination PDT with adoptive immunotherapy has been provided by Korbelik and Sun [110]. These authors attempted the transfer of a genetically altered natural killer cell line producing IL-2 in immuno-compromised mice bearing cervical squamous cell carcinoma, colorectal adenocarcinoma or mammary tumors. The combination was particularly effective when the adoptive transfer of the natural killer cell was performed immediately or shortly after PDT.

It has been observed that adoptive immunity can be transferred also through splenocytes cells. In fact, splenocytes derived from mice bearing colon carcinoma (that were pre-subjected to PDT), were particularly proficient in protecting naive animals from tumor development [111].

Another interesting combination of PDT with adoptive immunity has been offered by Korbelik and Dougherty, who demonstrated how photosensitizer activation could generate long-lasting tumor-sensitized immune [112]. In this work they noted that immuno-deficient mice recovered completely from mammary tumors, after receiving splenocytes from BALB/c donors, bearing the same tumor which was previously subjected to Photofrin/PDT.

### 3.4. Angiogenesis Inhibitors

Since angiogenesis promotes tumor growth and progression, its inhibition has been envisaged as a potentially effective anticancer strategy. PDT may induce direct vascular damage and subsequently a more extensive injury due to hypoxia that originates from the vascular obstruction. However, it has been also shown that PDT may act on the vasculature producing divergent effects in that the expression of some angiogenic factors (VEGF, COX-2 and Matrix Metalloproteinase) may be enhanced [113-115]. In any case, the eventual reperfusion of the previously occluded blood vessels may involve the formation of new blood vessels. These considerations and observations have suggested a combination of PDT with compounds that interfere with VEGF and/or COX-2 or their receptors as a workable therapeutic approach.

#### 3.4.1. VEGF/VEGF receptors

The simultaneous use of PDT and antiangiogenic agents has been described in several reports. For example [113], when the antiangiogenic peptides, IM862 or EMAP-II (VEGF inhibitors), were associated with Photofrin/PDT to cure a transplantable mammary carcinoma in mice, significant tumor regression and increased apoptosis was observed. The exclusive administration of anti-angiogenic agents or PDT alone was not as effective as the combination.

Ferrario and Gomer [116] studied the effect of Avastin, an antiangiogenic monoclonal antibody approved for the treatment of colon and rectal cancers in combination with Photofrin/PDT. Avastin was administered to tumor-bearing mice immediately after irradiation. The combination resulted in a statistically very significant increase in long-term tumor cures compared to individual treatments. Interestingly, the enhancement of anti-tumor activity was not obscured by undesired general toxicity of normal tissue.

The efficacy of Avastin has also been tested in combination with hypericin/PDT in bladder tumor xenografts. In these conditions the tumor responsiveness was improved as the expression of VEGF and other angiogenic proteins (angiogenin, bFGF, EGF, IL-6 and IL-8) was definitively reduced [117].

The combination of Photofrin/PDT with antiangiogenic monoclonal antibodies (MF1 and DC101) directed against VEGFR-1 and VEGFR-2, was found to be particularly effective in reducing the tumor size and in prolonging the survival time of nude mice bearing an experimental glioblastoma [118].

#### 3.4.2. COX-2

A negative loop has been reported in which PDT induces the expression of COX-2 [119] that in turn lessens the efficacy of PDT. Morevover, as COX-2 is frequently upregulated in cancers [120-122], the association of PDT with COX-2 inhibitors has been considered as an additional therapeutic strategy. For example, Ferrario *et al.* [123] made use of a combination of Celecoxib or NS-398 (COX-2 inhibitors) with Photofrin/PDT in an experimental mammary carcinoma. Both inhibitors, when administered *in vitro* after PDT, enhanced apoptosis, while the same combination *in vivo* decreased inflammation and reduced the expression of pro-angiogenic factors. Tumor-bearing mice treated with this combination exhibited significant improvement in long-term tumor-free survival when compared to animals treated with PDT or COX-2 inhibitors separately.

A few years ago, it was reported [124] that Rofecoxib, NS-398 and Nimesulide were not proficient in sensitizing colon carcinoma tumor cells to Photofrin/PDT-induced damage when COX-2 inhibitors were administered before PDT treatment. However, complete tumor response was achieved when COX-2 inhibitors were administered after PDT. The Authors concluded that the higher efficacy of PDT in association with COX-2 inhibitors was determined by the profound blood vessel damage induced by PDT accompanied by the simultaneous inhibition of neo-angiogenesis.

Akita *et al.* [125] investigated COX-2 expression and the inhibitory effects of Nimesulide in combination with 5-ALA/PDT in two human oral squamous cell carcinoma cell lines that profoundly differed in basal COX-2 expression levels. This paper pointed out that the effect of this combined treatment was effective only in cells overexpressing COX-2, as these cells represent a preferential target.

The upregulation of COX-2 after hypericin/PDT has been experimentally documented [126]. This overexpression was induced by the selective activation of the mitogen-activated protein kinase (MAPK) p38α and β at the protein and mRNA levels. Therefore, p38 MAPK inhibition was considered useful as additive therapy to suppress the expression of the mitogenic COX-2. Hendrickx and colleagues [127] exploited this concept, showing that the use of PD169316, a p38α MAPK inhibitor, improved the effectiveness of hypericin/PDT in curing human cervix carcinoma cells and human transitional cell carcinoma of the bladder. In the same study [127], the response to hypericin/PDT combined with either NS398 (COX-2 inhibitor) or PD169316 (p38 MAPK inhibitor) were compared. Although endothelial cell migration was impaired in both cases, inhibition of the p38α MAPK pathway was more effective in suppressing VEGF synthesis. Moreover, experiments including wild type or p38α knockout mouse embryonic fibroblasts clearly showed a propensity towards cell death in p38α-deficient cells that was not attainable through the use of NS398. Altogether, these results suggest that inhibition of p38 MAPK should be considered a more effective cancer treatment strategy than COX-2 inhibition.

#### 3.4.3. Metalloproteinases

Ferrario *et al.* [128] evaluated the anti-tumor activity of Photofrin/PDT followed by administration of Prinomastat, a potent synthetic metalloproteinase inhibitor, in a mouse mammary carcinoma. Tumors treated with Prinomastat alone exhibited a modest reduction in growth, but no decrease in tumor size or long-term cures. In contrast, the combination resulted in a significant difference in long-term cure rate compared to PDT alone.

The rationale of such an approach, at the moment much less exploited, resides in the strict relation linking the PDT-induced overexpression of metalloproteinases and angiogenesis [129].

#### 3.4.4. Other

5,6-dimethylxanthenone-4-acetic acid (DMXAA) is an agent currently undergoing clinical evaluation. It selectively causes the collapse of tumor vasculature leading to extensive cell death by altering tumor vascular permeability directly and indirectly, through the induction of various vasoactive mediators, such as TNF-α [130].

DMXAA has been shown to selectively enhance Photofrin/PDT activity against mouse tumors [131]. Bellnier *et al.* [131] noted that administration of low doses of DMXAA prior to PDT with Photofrin in a transplanted murine RIF-1 tumor model resulted in reduction of tumor size as well as in a significant delay in regrowth. However, the therapeutic efficacy of this combination was null if DMXAA was administered after PDT.

Similar findings have been recently reported by Seshadri and Bellnier [132] in a study of the effect of a combination of Photochlor/PDT and DMXAA in mice bearing colon carcinomas.

### 3.5. Receptor Inhibition

Many hormones and receptors and their downstream signaling pathways are often involved in cancer development and progression. Any component of cellular signaling that confers an advantage in cell growth must be considered as a potential target for cancer therapy. In this regard, particular attention should be paid to strategies aimed at blocking the receptors or their downstream effectors. Strategies that target hormone and growth factor receptors have been combined in selected cases with PDT. The choice of the target against a specific tumor requires a detailed knowledge of the characteristics of the specific cancer cells.

Tamoxifen has enjoyed considerable success in the treatment of breast cancer [133,134] and in general in other tumors overexpressing the estrogen-receptor. Tamoxifen has been successfully used in combination with Photofrin/PDT in human glioma cells *in vitro* [135].

More interestingly, Hydroxytamoxifen, a naturally occurring Tamoxifen metabolite, has been chemically linked to a porphyrin derivative (Pyropheophorbide). Indeed, it appears that the conjugate, maintaining its capacity to enter mammary tumor cells and to recognize its internal receptor, promoted selective photosensitizer accumulation with increased PDT efficacy [136].

The epidermal growth factor receptor (EGFR) is overexpressed in many different cancers and is currently seen as a promising target for cancer therapy [137].

Erbitux (Cetuximab), a chimeric human-murine monoclonal antibody, competitively binds to the extracellular domain of EGFR, inhibits dimerization and reduces cell proliferation, preventing metastasis and further tumor growth [138]. In most studies, the use of Erbitux in combination with chemotherapy and radiotherapy has demonstrated good tolerability and significant clinical efficacy [139]. Several authors have described the combination of EGFR inhibitors and PDT. For example, the anti-tumor effect of Erbitux in combination with hypericin/PDT has been studied in bladder carcinoma xenograft models [140]. In this paper, the authors concluded that the inhibition of EGFR expression combined with the effects of PDT strongly favored apoptosis and enhanced anti-tumor activity.

The combination of BPD/PDT with Cetuximab is another example of this association that has been described in a work dealing with ovarian cancer both *in vitro* and *in vivo* (xenograft models) [141]. The authors reported that combination therapy significantly reduced tumor growth and size and increased animal survival compared to monotherapy regimens. On the basis of these conclusions, they proposed the combination of BPD/PDT and Cetuximab as an alternative approach for the treatment of ovarian cancer in humans.

### 3.6. Radiotherapy

Radiotherapy (RT) is based on the use of ionizing radiation that works by damaging the DNA of cancerous cells. Undifferentiated malignant cells are considered more susceptible to radiation than normal cells as they reproduce more rapidly and have a diminished ability to repair sub-lethal DNA damage. The continuous accumulation of this damage eventually causes tumor cell death. Unfortunately, radiation is not completely selective, and injury to normal tissues and cells is virtually unavoidable.

Radiotherapy has exploited photosensitizers (principally porphyrins) to increase the intrinsic radiosensitivity of target cells [142-146]. However, during the last twenty years of research, RT has also been combined with PDT, although conflicting results have been reported. The first experiments in this area demonstrated that the association of Photofrin/ or 5-ALA/PDT [147,148] with radiation produced synergistic or antagonistic effects in critical dependence of the specific modalities and time intervals of administration of the therapeutic partners. More recently other Authors studying in several cancer model systems [149-153] the association of radiation with other photosensitizers such as HpD, Photosan, Photofrin and 5-ALA, described exclusively additive effects.

Very recently, the combination of 5-ALA/PDT and RT has been investigated in patients affected by Bowen's disease [154]. The research reported that the cure rate was improved by combination therapy. Compared to conventional RT, the observed synergistic effect allowed a strong reduction of the dose of radiation, thereby lessening important skin side-effects.

### 3.7. Miscellanea

#### 3.7.1. Proteasome inhibitors

Proteasome substrates include many signaling molecules, such as tumor suppressors, cell cycle regulators, transcription factors, anti-apoptotic proteins and others [155]. When the degradation of these proteins is halted, the effect is particularly felt by rapidly proliferating cells (as cancer cells), because their accelerated and uncontrolled proliferation rate can't be sustained for long [156].

Proteasome activity influences both the synthesis of NF-κB precursor and the degradation of NF-κB suppressor [157,158]. Therefore, inhibition of the proteasome may affect cancer progression by interfering with the pro-survival activity of NF-κB.

PDT and proteasome activity have been extensively studied in our laboratory in lung adenocarcinoma cells [159]. In these studies, we demonstrated that combination of Photofrin/PDT with proteasome inhibitors (Bortezomib or even aspirin) synergistically strengthens the overall therapeutic effect. Similar results were reported by others demonstrating in various cancer cell lines how Foscan/PDT in association with Bortezomib produced extensive cell death acting on the endoplasmic reticulum [160].

#### 3.7.2. Natural compounds

Several substances from natural sources including plants have been used in combination with PDT. As examples of this, we cite two very recent investigations that combined ceramides or curcumin with PDT.

Ceramides are normal components of the cell membrane. Besides structural functions, these lipid molecules, composed of sphingosine and fatty acids, are involved in many fundamental biological processes, such as regulation of cell differentiation, proliferation, apoptosis and senescence [161]. Separovic *et al.* [162] demonstrated that a ceramide analogue (C6-pyridinium ceramide) in combination with Photofrin/ or Foscan/PDT remarked improved overall long-term tumor cure in mouse squamous cell carcinoma models.

Curcumin is a compound extracted from herbs used in traditional Chinese medicine. In one study, Koon *et al.* [163] focused on both the intrinsic curcumin toxicity and photodynamic effect due to curcumin photosensitization. Exploiting the dual nature of curcumin, the authors demonstrated that exposure of nasopharyngeal cells to light resulted in advantage and, considering the reduced curcumin toxicity, hypothesized clinical use.

Table 2 summarizes the principal associations between the most popular photosensitizers and the various therapeutic partners (as detailed in individual paragraphs). Relative references are also indicated.

## 4. Conclusions

Numerous studies have documented the use of PDT strategies along with conventional cancer treatments to enhance antitumor response. The major part of the combined modality approaches proposed by the researchers that have been grossly summarized in this review, clearly lean towards increased therapeutic efficacy. Indeed, only some papers have really demonstrated synergy experimentally by means of appropriate calculation of Combination Indexes or isobolograms [13,14,167,168]. In any case, the hypothesis of cancer treatments founded on the simultaneous use of different molecular strategies emerges strengthened, and consequently suggest the use of PDT in combination with exploitable therapeutic resources. However, the dynamic nature of photosensitizer diffusion *in vivo* does not favor the selectivity between the tumor and normal tissue. This means that significant damage to non cancerous tissues during PDT can not be fully prevented. For this reason, a further way to improve combination therapy involving PDT implies a targeted delivery of the photosensitizer (and the drug) to the cancer site.

These targeted approaches along with systems (nanoparticles) designed to facilitate an appropriate distribution of the components of the combination therapy could finally bring PDT to be considered on the cutting edge of cancer therapy. Although the application of nanotechnologies to combined therapy is only beginning, it has already caught the attention of many researchers. It certainly deserves separate attention.

## Figures and Tables

**Table 1.** Photosensitizers approved in human diseases.

**Photosensitizer**	**Type of diseases**	**Country**
(*5*-*ALA*)	Actinic keratosis,	U.S., EU
5-aminolevulinate	Basal cell carcinoma	
Photofrin	Barrett's displasia	U.S., Canada, EU, UK
Photofrin	Cervical cancer	Japan
Photofrin	Endobronchial cancer	Canada, Most EU Countries, Japan, U.S.
Photofrin	Esophageal cancer	Canada, Most EU Countries, Japan, U.S.
Photofrin	Gastric cancer	Japan
Photofrin	Papillary bladder cancer	Canada
Foscan	Head and neck cancer	EU, Norway, Iceland
Verteporfin	Age-related Macular Degeneration	Canada, Most EU Countries, Japan, U.S.

**Table 2.** Principal types of associations and experimental systems.

**Photosensitizer**	**in association with**	**Experimental system**	**Ref.**
**Photofrin**	**Cisplatin** DNA-alkylating agent *(chemotherapeutic agent)*	mouse lymphoma cells esophageal carcinoma cells	[29,30]
	**Mitomycin C** DNA synthesis inhibitor *(chemotherapeutic agent)*	h-colon adenocarcinoma cells murine fibrosarcoma rat colon carcinoma	[41-44]
	**Gemcitabine** DNA base analogue *(chemotherapeutic agent)*	h-adenocarcinoma non small cell lung cancer cells	[55]
	**Vincristine** mitosis inhibitor *(chemotherapeutic agent)*	ovarian cancer cells	[61]
	**G-CSF** granulocyte colony stimulating factor *(immunotherapy)*	colon adenocarcinoma (mice) Lewis lung tumor (mice)	[75]
	**DBPMAF** macrophage activating factor *(immunotherapy)*	squamous cell murine model	[79]
	**IM862 and EMAP-II peptides** VEGF inhibitors *(antiangiogenic therapy)*	mammary carcinoma (mice)	[113]
	**Avastin** mAb that binds VEGF-A *(antiangiogenic therapy)*	colon and rectal cancers	[116]
	**MF1 and DC101** mAb anti VEGFR-1/VEGFR-2 *(antiangiogenic therapy)*	glioblastoma (mice)	[118]
	**Celecoxib and NS-398** COX-2 inhibitors *(antiangiogenic therapy)*	mammary carcinoma (mice)	[123]
	**Prinomastat** metalloproteinse inhibitor *(antiangiogenic therapy)*	mouse mammary carcinoma	[128]
	**DMXAA** tumor vasculature collapse inhibitor *(antiangiogenic therapy)*	colon carcinomas (mice) murine radiation induced fibrosarcoma	[131,132]
	**Tamoxifen** EGFR antagonist *(receptor inhibition)*	h-glioma cells	[135]
	**Bortezomib** proteasome inhibitor *(targeted approach)*	h-non small cell lung cancer cell lines	[159]
	**C6-pyridinum ceramide** ceramide analogue *(natural compound)*	mouse squamous cell carcinoma	[162]
**Indocyanine Green**	**Cisplatin** DNA-alkylating agent *(chemotherapeutic agent)*	h-breast cancer cells	[14]
**Hematoporphyrin Derivative**	**Cisplatin** DNA-alkylating agent *(chemotherapeutic agent)*	bladder cancer cells urothelial cells	[31]
	**Corynebacterium parvum derivative** immunoadjuvant agent *(immunotherapy)*	bladder cancer (mice)	[90]
	**OK432** streptococcus piogenes derived *(immunotherapy)*	Mouse squamous cell carcinoma	[94]
	**butyl-4-hydroxyanisole** *(anti oxidant agent)*	Ehrlich scites carcinoma cells	[23]
**5-δ-Aminolevulinic Acid**	**Doxorubicin** DNA intercalating / topoisomerase II inhibitor *(chemotherapeutic agent)*	mammary adenocarcinoma (mice)	[35]
	**Mitomycin C** DNA synthesis inhibitor *(chemotherapeutic agent)*	bladder tumors h-bladder tumor	[46,47]
	**Methotrexate** DNA synthesis inhibitor (chemotherapeutic agent)	h-prostate carcinoma cells epithelial squamous carcinoma cells	[58,59]
	**Imiquimod** Induction of cytokines *(immunotherapy)*	Bowenoid papulosis patients	[76]
	**Nimesulide** COX-2 inhibitor *(antiangiogenic therapy)*	h-oral squamous cell lines	[125]
	**Ionizing radiation** (*radiotherapy)*	Bowen's disease patients	[154]
	**Ascorbate** *(anti oxidant agent)*	rat sarcoma cancer cells	[21]
**Al-Phtalocyanine**	**Doxorubicin** DNA intercalating / topoisomerase II inhibitor *(chemotherapeutic agent)*	leukemia and lymphoma (mice)	[36]
	**Etoposide** topoisomerase II inhibitor (*chemotherapeutic agent)*	h-leukemia cells	[53]
	**Bacille Calmette-Guerin** immunoadjuvant (*immunotherapy*)	mammary sarcoma (mice)	[91,92]
**Foscan / mTHPC**	**Doxorubicin** DNA intercalating / topoisomerase II inhibitor *(chemotherapeutic agent)*	murine hepatoma cells	[38]
	**Mitomycin C** DNA synthesis inhibitor (*chemotherapeutic agent*)	murine fibrosarcoma	[45]
	**Vincristine** mitosis inhibitor *(chemotherapeutic agent)*	murine mammary cancers	[60]
	**Bacille Calmette-Guerin** immunoadjuvant *(immunotherapy)*	mammary sarcoma (mice)	[91,92]
	**NK cells producing IL-2** *(adoptive immunotherapy)*	cervical squamous cell, mammary, colorectal adeno-carcinoma (mice)	[110]
	**C6-pyridinum ceramide** x(*natural compound)*	mouse squamous cell carcinoma	[162]
	**α-tocopherol** *(antioxidant)*	h-colon adenocarcinoma cells	[24,25]
**Chlorins (various)**	**Irinotecan** topoisomerase I inhibitor *(chemotherapeutic agent)*	h-colon xenografts	[51]
	**Doxorubicin** DNA intercalating / topoisomerase II inhibitor *(chemotherapeutic agent)*	ovarian carcinoma (mice)	[37]
	**Bacille Calmette-Guerin** immunoadjuvant (immunotherapy)	mammary sarcoma (mice)	[91,92]
**Photosan**	**Gemcitabine** DNA base analogue *(chemotherapeutic agent)*	h-pancreatic cancer (mice)	[56]
**Verteporfin Benzoporphyrin Derivative**	**paclitaxel** mitosis inhibitor *(chemotherapeutic agent)*	h-gastrointestinal tumor	[64]
	**G-CSF** granulocyte colony stimulating factor*(immunotherapy)*	murine squamous cell carcinoma (mice)	[74]
	**Zymosan** complement activator *(immunotherapy)*	malignant mouse melanoma	[86]
	**γ-inulin** complement activator (immunotherapy)	malignant mouse melanoma	[86]
**Verteporfin Benzoporphyrin Derivative**	**IFN-γ** immunoregolatory cytokine *(immunotherapy)*	malignant mouse melanoma	[86]
	**Bacille Calmette-Guerin** Immunoadjuvant (immunotherapy)	mammary sarcoma (mice)	[91,92]
	**Cyclophosphamide** reduction of immunosoppressive T cells *(immunotherapy)*	metastic murine model	[99-101]
**Hypericin**	**Avastin** mAb that binds VEGF-A *(antiangiogenic therapy)*	bladder tumor xenografts	[117]
	**PD169316** p38 MAPK inhibitor / COX-2 inhibitor (antiangiogenic therapy)	h-cervix carcinoma cell lines h-bladder transitional cell carcinoma	[127]
	**Erbitux** EGFR antagonist *(receptor signalling inhibition)*	bladder carcinoma xenografts	[140]
